# Exosomal miR-214-3p from senescent osteoblasts accelerates endothelial cell senescence

**DOI:** 10.1186/s13018-023-03859-6

**Published:** 2023-05-30

**Authors:** Zhen Guo, Jing Li, Jiyong Tan, Sainan Sun, Qing Yan, Hao Qin

**Affiliations:** 1 Department of Orthopedics, Guigang City People’s Hospital, No. 1 Zhongshan Middle Road, Gangbei District, Guigang, 537100 Guangxi People’s Republic of China; 2 Department of Physiology, Guangxi Medical University, Nanning, People’s Republic of China; 3 Key Laboratory of Longevity and Aging-Related Diseases, Ministry of Education, Guangxi Medical University, Nanning, People’s Republic of China

**Keywords:** miR-214-3p, Osteoblast, Endothelial cell, Senescence, Exosome

## Abstract

**Background:**

Osteoporosis is a common systemic bone disease that leads to bone fragility and increases the risk of fracture. However, the pathogenesis of osteoporosis is considered to be highly complex. The exosomes can regulate the communication between cells. The specific mechanism of information transmission between osteoblasts and endothelial cells is worthy of further study.

**Methods:**

Exosomes were isolated and verified from senescent osteoblasts. The source and properties of exosomes were determined by TEM, particle size analysis and western blot. We established the co-culture model of endothelial cells and senescent osteoblasts. We used qRT-PCR to identify differentially expressed miRNAs. The functional changes of vascular endothelial cells were verified by cell transfection. β-Galactosidase cell senescence assay, Hoechst cell apoptosis assay, Ki67 cell proliferation assay and Transwell migration assay were used to verify cell senescence, apoptosis, proliferation, and migration. The potential target gene of miRNA was detected by bio-informatics pathway and double luciferase report.

**Results:**

We discovered that senescent osteoblasts could promote the senescence and apoptosis of vascular endothelial cells and inhibit their proliferation and migration. miR-214-3p was upregulated in senescent osteoblast-derived exosomes. miR-214-3p could effectively promote senescence and apoptosis of endothelial cells and inhibit proliferation and migration ability. L1CAM is a miR-214-3p direct target gene determined by bio-informatics and double luciferase report.

**Conclusions:**

In conclusion, senescent osteoblast-derived exosomes can accelerate endothelial cell senescence through miR-214-3p/L1CAM pathway. Our experiments reveal the role of exosomes in the skeletal microenvironment.

## Introduction

Osteoporosis is becoming prevalent as the proportion of the elderly population increases. The typical characteristics of osteoporosis are low bone mass or bone mineral density, destruction of bone microstructure, increased brittleness, and easy fracture [1, 2]. Bone has an extensive network of blood vessels and capillaries, which provide essential oxygen and nutrients for the formation and development of bone. Different signaling pathways mediate bone formation and development between bone cells and endothelial cells [3, 4]. More importantly, there are changes in the number of osteoblasts and decreases in angiogenesis in the elderly bone tissue suffering from bone loss [5]. miRNAs, as a small endogenous noncoding RNA, restrain gene expression at post-transcriptional regulation of mRNA by binding to the 3'-UTR or open reading frame region of the target mRNA [6, 7]. miR-214-3p has been linked with the development and progression of cancer disease [8], cardiovascular disease [9, 10], renal disease [11], and bone metabolism disease [12]. However, there is a lack of functional miR-214-3p identified as intercellular signals between osteoblasts and endothelial cells in bone microenvironment.

Previous studies have confirmed that the exosomes mediate miRNAs to participate in cell-specific communication [13]. Exosomes are expected to become a reliable new method for the treatment of osteoporosis [14]. Due to the vital function of miR-214 on osteoblasts and endothelial cells [9, 15–17], we explored whether miR-214-3p from senescent osteoblast-derived exosomes could accelerate endothelial cell senescence in the present study.

## Methods

### Cell culture and transfection

Primary osteoblasts were derived from 5 to 7-day-old newborn BALB/c mice (Animal Center, Guangxi Medical University, China). Collected bone tissues were digested using Type II Collagenase (Gibco, CA, USA). Vascular endothelial cells were derived from cell lines (MPVEC, Aolu Company, Shanghai, China). Primary osteoblasts and vascular endothelial cells were cultured, respectively, in MEMα medium (Gibco, CA, USA) and DMEM medium (Gibco, CA, USA) supplemented with 10% fetal bovine serum (Gibco, CA, USA) and 100 U/ml each of penicillin and streptomycin (Gibco, CA, USA). Then, cells were maintained at 37 ℃ in a thermostatic incubator with 5% CO_2_. D-gal (20 g/L, Gibco, CA, USA) was added to the medium to induce cellular senescence. Osteoblasts were added to the upper chamber of a Transwell chamber (Corning, USA) and adjusted to a 2.0 × 10^5^ cells/ml density. Endothelial cells were inoculated in six-well plates (Corning, USA) at a 1.0 × 10^5^ cells/ml density. Put the Transwell chambers into the plates after both cell types reached 50% adhesion growth density to establish the co-culture model. miR-214-3p mimic and inhibitor with their negative controls were purchased from RiboBio company. According to the manufacturer’s protocol, they were transfected into the endothelial cells utilizing riboFECT CP reagents (RiboBio, Guangzhou, China).

### Exosomes isolation and transmission electron microscopy (TEM)

SBI ExoQuick-TC ULTRA EV Isolation Kit (System Biosciences, USA) was used to isolate exosomes from osteoblasts culture supernatant. The isolated exosomes were resuspended in PBS, and 5 μL of the diluted mixture was dropped onto sealing films. Put the carbon-coated copper mesh on the droplets floating. Subsequently, the sealing film was stained with 20 μl of 1% phosphotungstic acid-staining droplet. After drying naturally, a standard TEM with a Philips CM120 microscope was used to observe exosomes and take a picture.

### Nanoparticle analysis

Phosphate-buffered saline was used to dilute the extract exosomes. A vortex mixer was used to mix the exosomes evenly. The exosomes were measured with Nanosight NS300 (Malvern, Britain). Each sample was measured three times and 30 s each time.

### Western blot

The samples were lysed in RIPA buffer (Thermo Fisher Scientific, USA). The proteins were isolated in 10% SDS polyacrylamide gel electrophoresis and then transferred onto PVDF membranes. Membranes were blocked and cultivated with primary antibodies overnight at 4 °C. The primary antibodies include CD9, CD63, TSG101, and calnexin (all from Abcam). Membranes were washed and further incubated with either HRP-labeled sheep anti-rabbit secondary antibodies or HRP-labeled sheep anti-mouse secondary antibodies for 2 h at room temperature and washed again. Protein bands were detected with the enhanced chemiluminescence detection system (Millipore, Bedford, MA).

### RNA isolation, reverse transcription, and quantitative real-time polymerase chain reaction (qRT-PCR)

TRIzol reagent (Invitrogen, CA, USA) was used to isolate total RNA from the cells following the manufacturer’s guidelines. The RNA was converted into cDNA by reverse transcription using the microRNA First-Strand cDNA Synthesis Kit (Sangon Biotech, Shanghai, China) and RevertAid First Strand cDNA Synthesis Kit (Sangon Biotech, Shanghai, China). Real-time PCR was performed to detect the RNA expression using SYBR Green PCR Master Mix (Applied Biosystems) and running with the Applied Biosystems 7300 Fast Real-Time PCR System (Applied Biosystems). The miRNA expression level was standardized to U6, and the mRNA expression level was standardized to GAPDH. The results were analyzed by the 2^−△△Ct^ method. The primer sequences used in this study are described in Table.1.

**Table 1:** Primer sequences used in this study

Primer name	Primer sequence
GAPDH	F: 5′-GGTTGTCTCCTGCGACTTCA-3′
	R: 5′-TGGTCCAGGGTTTCTTACTCC-3′
LICAM	F: 5′-TGCTCCTCATCCTGCTCATCCTC-3′
	R: 5′-TCACTGTACTCGCCGAAGGTCTC-3′
mmu-miR-214-3p	F: 5′-TACAGCAGGCACAGACAGGC-3′

### Cell senescence assay

Senescence-associated-β-galactosidase (SA-β-gal) staining was performed to detect cell senescence using a Cell Senescence β-Galactosidase Staining Kit (Beyotime, Shanghai, China) according to the manufacturer’s protocol. When the cell density reached about 50% under a standard optical microscope, the cells were fixed and stained with SA-β-gal staining solution at 37 °C overnight. After washing the cells with PBS, a digital microscope camera (Olympus) was used to capture images (200 ×). Results were expressed as the percentage of SA-β-gal-positive cells among the total cells counted.

### Cell apoptosis assay

A Hoechst 33,258 Staining Kit (Beyotime, Shanghai, China) was used to evaluate the cell nuclear apoptotic changes. Cells were fixed, washed, and stained according to the manufacturer’s instructions. A drop of anti-fluorescence sealing liquid was placed on the cells. The blue-stained nucleus could be detected, and the images (200 ×) were taken under an inverted fluorescence microscope (Olympus, Japan).

### Cell proliferation assay

Cell proliferation was detected by the SP staining kit (Zhongshan Jinqiao, Beijing, China) and the Ki67 cell proliferation detection kit (Sangon Biotech, Shanghai, China). Cells were used when they reached a confluence of 70%. The cells were incubated with the antibodies after they were immobilized on glass slides, and then, they were added onto the climbing tablet to carry out DAB and hematoxylin staining. Photographs (200 ×) were taken after the cells were sealed.

### Cell migration assay

Transwell chambers (8 μm, Corning, USA) were applied following the manufacturer’s protocol for the cell migration experiment. The cell density was adjusted to 2 × 10^5^ cells/ml. Subsequently, 500 μl serum-free DMEM with transfected cells was added to the upper chambers, and the lower chamber was filled with a 750 μl medium containing serum. After 12-h incubation, the migrated cells were fixed with POM and stained with 500 μ1 0.1% crystal violet solution for 30 min. Then, an inverted microscope was used to count the number of migrated cells and take the photographs (200 ×).

### Dual-Luciferase assay

The L1CAM 3′-UTR wild-type (WT) and mutated (MUT) sequences of the target gene L1CAM, were cloned into the pSICheck-2 plasmid. Next, 293 T cells were cultured using 96-well plates. Objective plasmids and mutant plasmids were mixed with miR-214-3p mimic and scrambled RNA as a negative control. Lipofectamine 2000 transfection reagent was used for transfection. After transfected 48 h, the relative luciferase activity was detected using Dual-Luciferase System Kit (Promega, USA) following the manufacturer’s instructions.

### Statistical analysis

All data were presented as mean ± S.D. All statistical analyses for this research were executed by SPSS 23.0 software (Chicago, IL, USA) and GraphPad Prism 8 software (La Jolla, CA, USA). The significance of the difference in the mean was estimated using a t-test and one-way analysis of variance. For all analyses, * was used to represent *p* < 0. 05, while ** was used to represent *p* < 0. 01.

## Results

### Senescent osteoblast-derived exosomes promoted senescence and apoptosis of endothelial cells

**Fig. 1:**
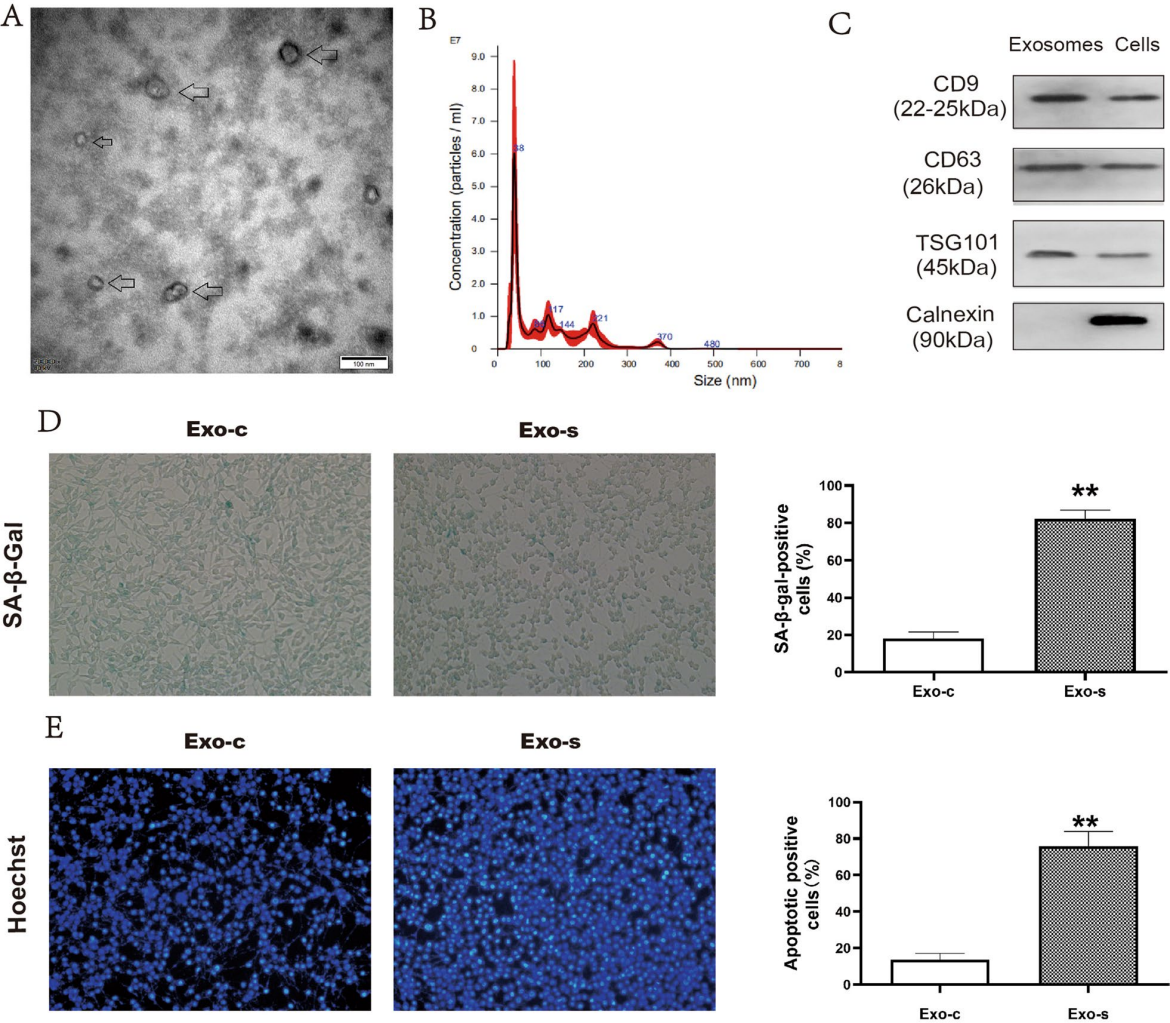
Effect of senescent osteoblast-derived exosomes on the biological function of endothelial cells. **A** Electron microscope scanning of exosome. **B** Particle size analysis of exosome. **C** Western blot analysis of exosome-enriched proteins (CD9 (22–25 kDa), CD63 (26 kDa) and TSG101 (45 kDa)) and the key protein for miRNA function (Calnexin (90 kDa)). **D** β-Galactosidase senescence staining of vascular endothelial cells co-cultured with the exosomes derived from control (Exo-c) or senescence (Exo-s) osteoblastic cells. **E** Hoechst apoptosis staining of vascular endothelial cells co-cultured with the exosomes derived from control (Exo-c) or senescence (Exo-s) osteoblastic cells (∗∗*p* < 0.01)

Exosomes were extracted from senescent osteoblasts and analyzed by transmission electron microscope and particle size (Fig. 1A, B). The western blot method further confirmed that the exosome marker proteins were enriched in exosomes (Fig. 1C). After treatment of senescent osteoblast-derived exosomes (Exo-s), senescence and apoptosis of vascular endothelial cells increased significantly (Fig. 1D, E). These indicated that senescent osteoblasts could accelerate endothelial cell senescence and apoptosis through the exosome pathway.

### Senescent osteoblasts affected endothelial cell functions through exosome-mediated miR-214-3p

**Fig. 2:**
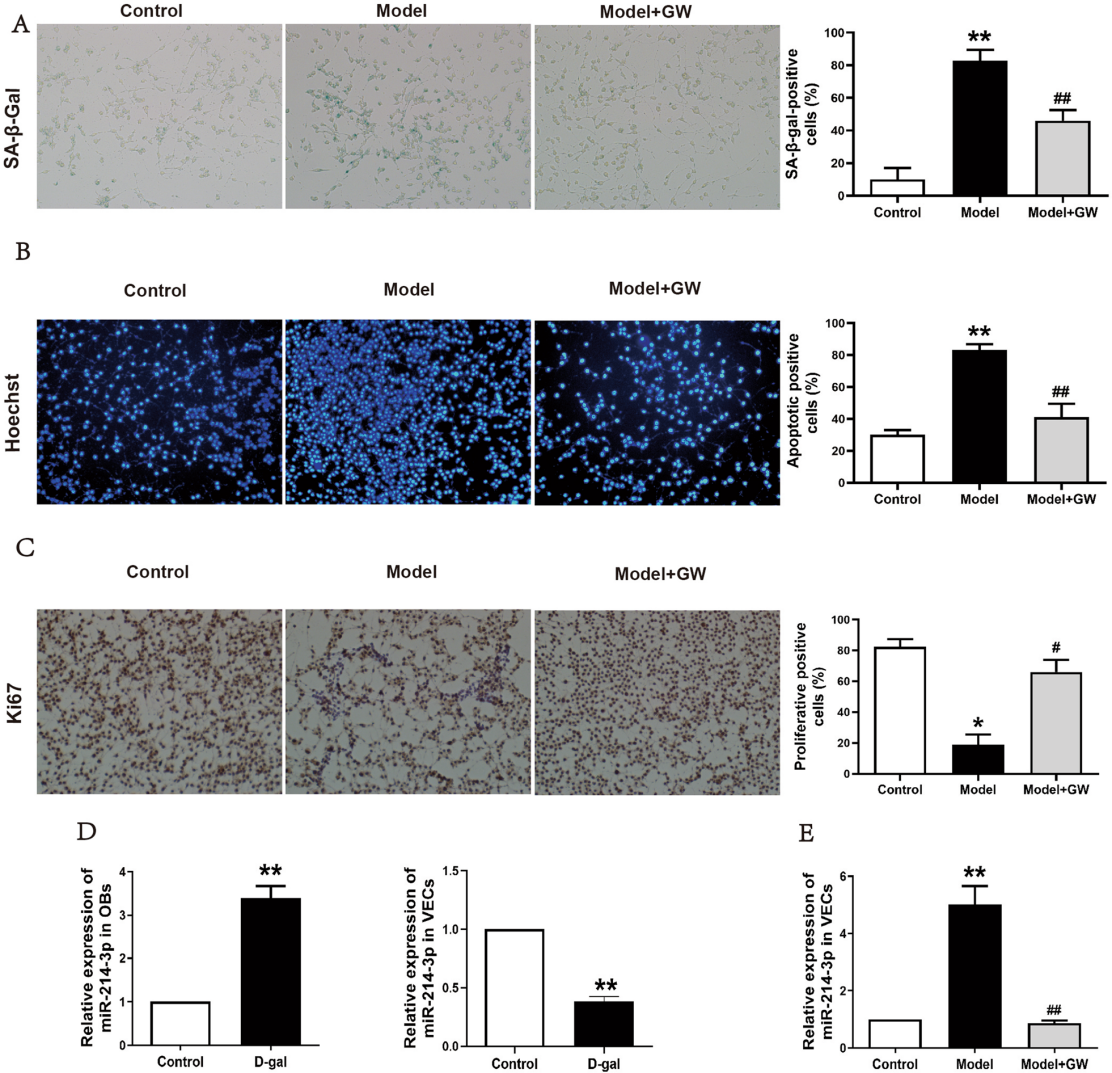
The biological function of endothelial cells and expression of miR-214-3p in co-culture system. **A** β-Galactosidase senescence staining of endothelial cells after co-cultured with normal osteoblasts (Control) or senescent osteoblasts (Model) and GW4869 exosome inhibitor (Model + GW). **B** Hoechst apoptosis staining of endothelial cells after co-cultured with normal osteoblasts (Control) or senescent osteoblasts (Model) and GW4869 exosome inhibitor (Model + GW). **C** Ki67 proliferation staining of endothelial cells after co-cultured with normal osteoblasts (Control) or senescent osteoblasts (Model) and GW4869 exosome inhibitor (Model + GW). **D** Expression of miR-214-3p in senescent osteoblasts (OBs) and senescent vascular endothelial cells (VECs). **E** Expression of miR-214-3p in vascular endothelial cells after co-cultured with normal osteoblasts (Control) or senescent osteoblasts (Model) and GW4869 exosome inhibitor (Model + GW). (∗ means comparison with the control group, *p* < 0.05; # means comparison with the model group, *p* < 0.05; ∗∗ means comparison with the control group, *p* < 0.01; ## means comparison with the model group, *p* < 0.01)

In the co-culture system, the endothelial cells co-cultured with senescent osteoblasts occurred in promotion of cellular senescence and apoptosis and inhibition of proliferation (Fig. 2A, B, and C). In contrast, GW4869 exosome inhibitor could decrease the senescence and apoptosis-positive rate and increase the proliferation rate of endothelial cells in the co-culture system (Fig. 2A, B, and C). Furthermore, the qRT-PCR results indicated that the expression of miR-214-3p increased in senescent osteoblasts but decreased in senescent endothelial cells (Fig. 2D). However, the relative expression was found to have increased in vascular endothelial cells co-cultured with senescent osteoblasts (Fig. 2E). And compared with the control group, there was no significant change in miR-214-3p expression level of endothelial cells after senescent osteoblasts treated with GW4869 exosome inhibitor (Fig. 2E). These suggested the senescent osteoblasts could regulate endothelial cell functions through exosome-mediated miR-214-3p.

### miR-214-3p promoted endothelial cell senescence and apoptosis while suppressed proliferation and migration

**Fig. 3:**
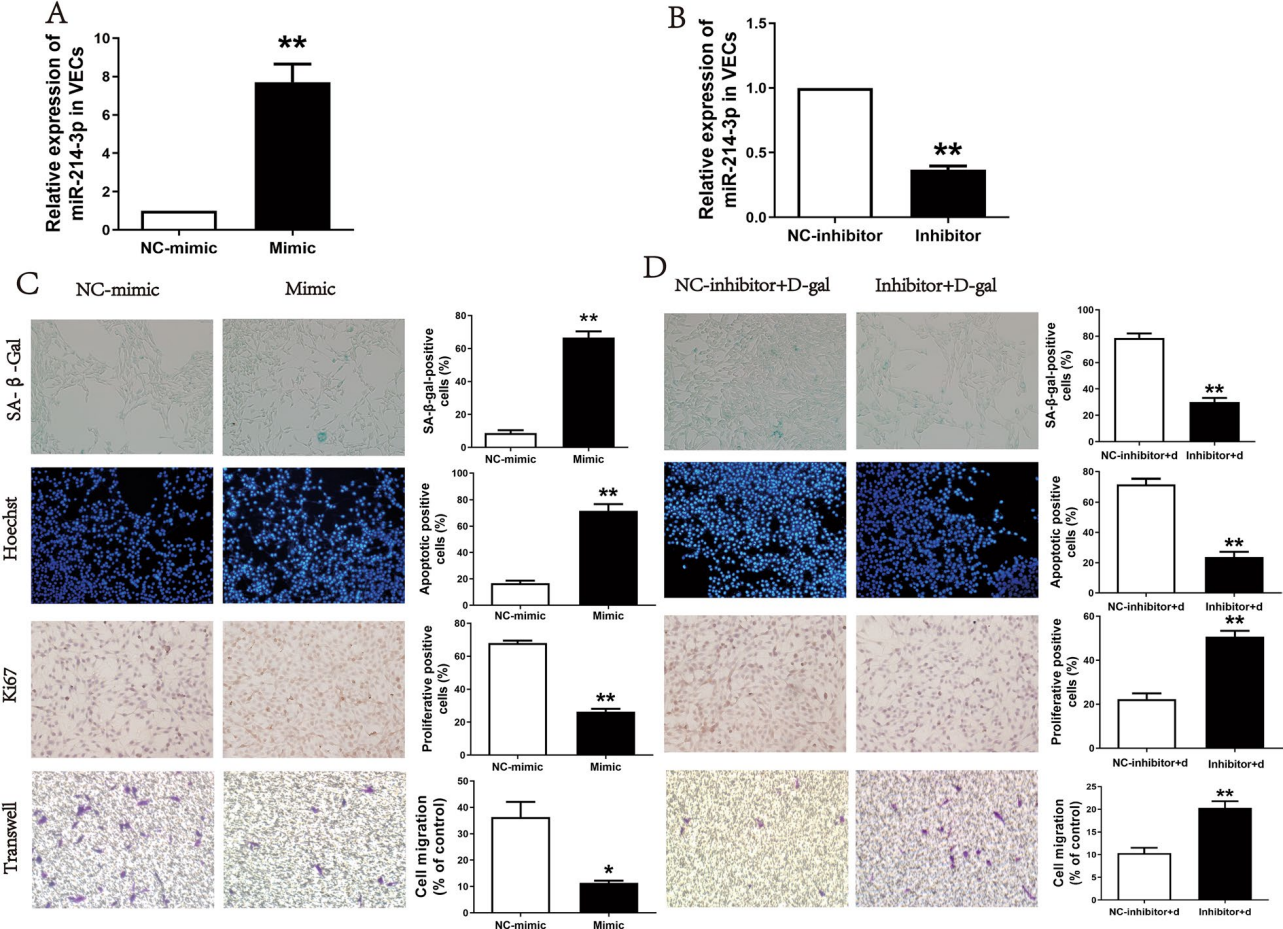
Effects of miR-214-3p on the senescence, apoptosis, proliferation and migration of endothelial cells. **A** Expression of miR-214-3p after transfection with miR-214-3p mimic in endothelial cells. **B** Expression of miR-214-3p after transfection with miR-214-3p inhibitor in endothelial cells. **C** β-Galactosidase senescence staining, Hoechst apoptosis staining, Ki67 proliferation staining and Transwell migration of vascular endothelial cells after miR-214-3p mimic treatment. **D** β-Galactosidase senescence staining, Hoechst apoptosis staining, Ki67 proliferation staining and Transwell migration of senescent vascular endothelial cells after miR-214-3p inhibitor treatment (∗*p* < 0.05; ∗∗*p* < 0.01)

After miR-214-3p mimic or inhibitor was transfected into endothelial cells, the transfection efficacy was tested by qRT-PCR (Fig. 3A and 3B). The SA-β-gal staining and Hoechst apoptosis staining were used to investigate the potential influence of miR-214-3p on endothelial cell functions. The results showed that after miR-214-3p mimic treatment, the senescence and apoptosis-positive rate increased in endothelial cells (Fig. 3C). However, the senescence and apoptosis-positive rate decreased in senescent endothelial cells treated by miR-214-3p inhibitor (Fig. 3D). Meanwhile, Ki67 proliferation staining showed that miR-214-3p overexpression could inhibit endothelial cell proliferation (Fig. 3C). The Transwell assay confirmed that miR-214-3p overexpression could attenuate the migration ability of endothelial cells (Fig. 3C). In contrast, the inhibition of miR-214-3p expression enhanced senescent endothelial cell proliferation and migration ability (Fig. 3D).

### L1CAM is a potential target gene of miR-214-3p

**Fig. 4:**
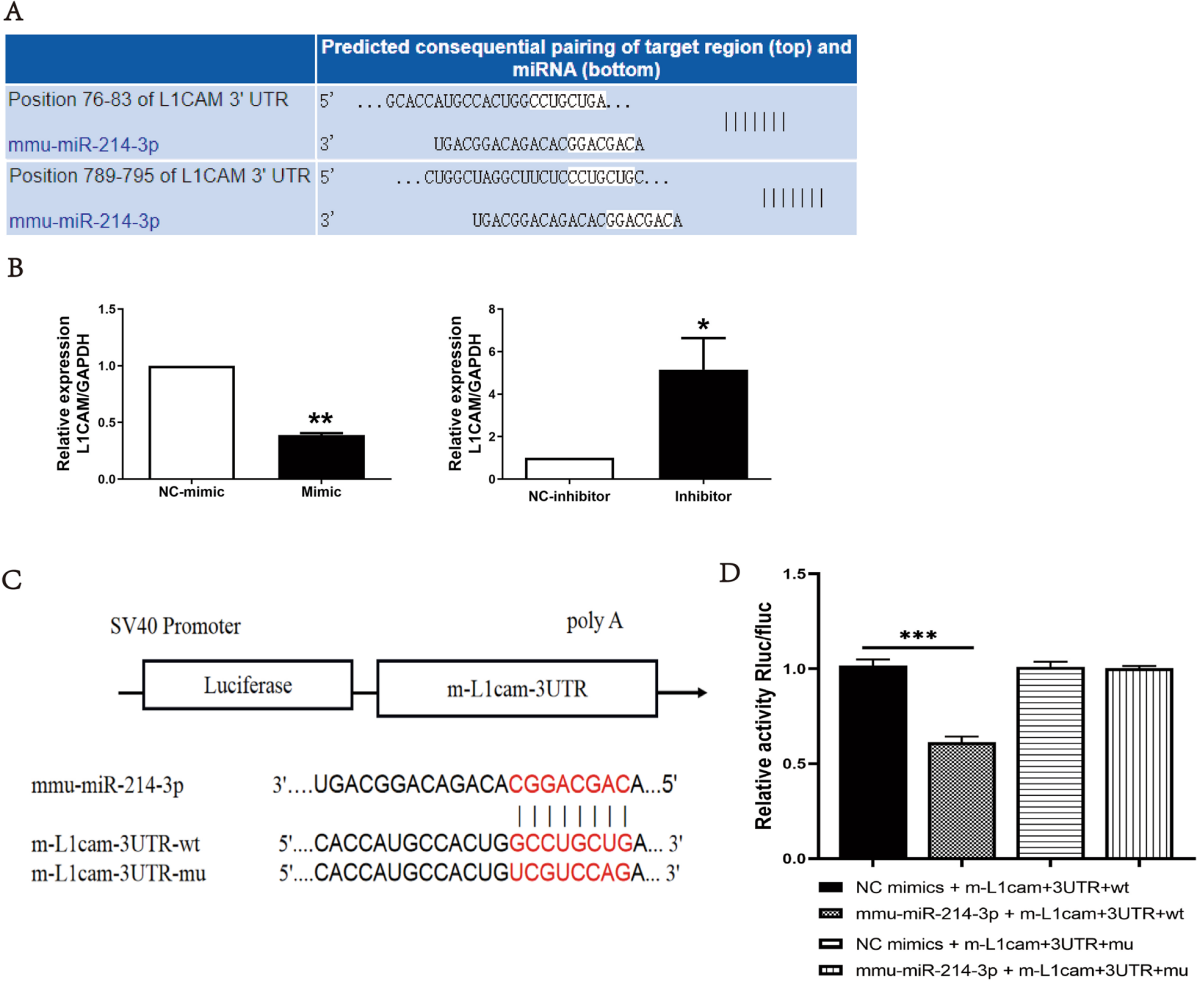
L1CAM as a potential target of miR-214-3p. **A** Identify potential target gene L1CAM by TargetScan bio-informatics database (This figure is from the TargetScan website). **B** Expression of L1CAM mRNA in endothelial cells after transfected with miR-214-3p mimic or inhibitor. **C** The predicted binding site of L1CAM targeted by miR-214-3p. **D** The dual-luciferase assay verified the targeted relationship between L1CAM and miR-214-3p (∗*p* < 0.05; ∗∗*p* < 0.01; ∗∗∗*p* < 0.001)

A potential target gene of miR-214-3p, L1CAM, was screened out using TargetScan bio-informatics database (Fig. 4A). The expression of L1CAM mRNA was significantly decreased in the endothelial cells with miR-214-3p overexpression, while significantly increased in the endothelial cells with low miR-214-3p expression (Fig. 4B). The double luciferase report showed that the fluorescence activity of L1CAM was significantly decreased in the miR-214-3p/WT co-transfection group. In contrast, the fluorescence activity of L1CAM was no different between NC group and the miR-214-3p/MUT co-transfection group (Fig. 4C, D). These indicated that L1CAM was a direct target gene of miR-214-3p.

## Discussion

Exosome-mediated transfer of miRNA plays an essential role in regulating the bone microenvironment [18]. Research has shown that exosomes participate in bone homeostasis by regulating the differentiation and activity of bone cells including mesenchymal stem cells, osteoblasts and osteoclasts [14]. In our study, we found that senescent osteoblasts could accelerate endothelial cell senescence through exosome-mediated miR-214-3p. Previous studies have uncovered the critical role of endothelial cell-dependent osteoblastic bone formation and degeneration [19–21]. These revealed the effect of senescent osteoblasts on endothelial cells might accelerate osteoporosis during bone pathological change process via cell–cell communication.

In recent years, there has been increasing research on the role of noncoding RNAs in musculoskeletal disorders. It has been found that miRNAs play an important role in the physiopathology, diagnosis, and treatment of osteoarthritis through interactions with multiple target genes [22]. Small interfering RNAs can be used to identify molecular targets of rheumatoid arthritis and evaluate the efficacy of specific drugs, thereby contributing to the treatment of rheumatoid arthritis [23]. Small interfering RNAs can participate in the tendon repair process and can be used to study and identify possible therapeutic targets in tendon healing [24], especially that miRNAs have been found to have therapeutic potential in the repair of tendon injuries [25].

In our previous study, we analyzed the miRNA expression profile of bone tissue in aging mice, and found differentially expressed miRNAs that contain miR-214-3p. What’s more, miR-214-3p has been reported to be highly variable in its expression level in multiple bone metabolic diseases. miR-214-3p inhibits BMP/Smad signaling pathway to delay fracture healing in rats with osteoporotic fracture [26]. miR-214-3p derived from osteoclastic exosome inhibits osteoblastic bone formation [12]. Our study found that miR-214-3p overexpressed in senescent osteoblasts could promote endothelial cell senescence via exosome pathway. These indicated that the changes in blood vessels may affect the function of bone cells, thereby accelerating degenerative bone diseases.

Meanwhile, accompanied with the cellular senescence, miR-214-3p mediated by osteoblast exosomes could inhibit proliferation and migration of endothelial cells in our study. However, previous study showed that miR-214-3p derived from chondrocytes promoted endothelial cell angiogenesis and migration via paracrine VEGF [15]. These differences are probably due to various cellular physiological condition induce different gene expressions, which occur in diversified miRNAs functions.

In the present study, we also demonstrate that the L1 cell adhesion molecule (L1CAM) is the direct target gene of miR-214-3p. We mainly use TargetScan and other bio-informatics databases to find the target genes of miR-214-3p. We found L1CAM has a close relationship with endothelial cells in the genes we screened. L1CAM is a cell surface glycoprotein with an extracellular portion [27], which plays a pro-angiogenic role in endothelial cells of tumor-related vessels [28]. For example, L1CAM promoted STAT3 activation via the IL-6/IL-6Rα axis to regulate tumor angiogenesis and vessel stabilization [28]. Moreover, the lack of L1CAM may be associated with cellular senescence, apoptosis, and proliferation [29, 30]. These show that L1CAM is an essential gene that regulates endothelial cell functions. In addition, the overexpression of miR-214-3p could promote atherosclerosis by directly targeting ATG5 [9]. Inhibition of miR-214-3p protected endothelial cells from ox-LDL-induced damage by targeting GPX4 [31]. All these findings further suggested miR-214-3p could regulate endothelial cell functions by targeting L1CAM, which may affect the angiogenesis and structure of blood vessels in the bone microenvironment and accelerate bone degenerative process.

Our study provides new findings on senescent osteoblasts can regulate the biological function of vascular endothelial cells. These findings perfect the mechanism of osteoblasts and endothelial cells interact with each other in bone microenvironment during senescence. This study elucidates a novel cellular and molecular pathway that influences miR-214-3p expression, and transfer between different cell types could lead to the development of new therapeutic approaches and new insights in bone aging pathophysiology. Therapies targeting miR-214-3p may serve as an effective treatment for bone regenerating patients. It provides targets for the treatment of osteoporosis and other bone-related diseases, and in the future, bone-related diseases can be treated clinically by designing target-specific drugs.

## Conclusion

Taken together, senescent osteoblast-exosomal miR-214-3p may accelerate endothelial cell senescence by targeting L1CAM. miR-214-3p may be an essential therapeutic target for osteoblasts and endothelial cells on aging-related osteoporosis.

## Data Availability

The datasets generated or analyzed during the current study are included in the manuscript submission.

## References

[1] Johnston CB, Dagar M (2020). Osteoporosis in older adults. Med Clin North Am.

[2] Bliuc D, Alarkawi D, Nguyen TV, Eisman JA, Center JR (2015). Risk of subsequent fractures and mortality in elderly women and men with fragility fractures with and without osteoporotic bone density: the Dubbo Osteoporosis Epidemiology Study. J Bone Miner Res.

[3] Hankenson KD, Dishowitz M, Gray C, Schenker M (2011). Angiogenesis in bone regeneration. Injury.

[4] Kusumbe AP, Ramasamy SK, Adams RH (2014). Coupling of angiogenesis and osteogenesis by a specific vessel subtype in bone. Nature.

[5] Wei Y, Sun Y (2018). Aging of the bone. Adv Exp Med Biol.

[6] Correia de Sousa M, Gjorgjieva M, Dolicka D, Sobolewski C, Foti M (2019). Deciphering miRNAs' Action through miRNA Editing. Int J Mol Sci.

[7] Yang K, Shi J, Hu Z, Hu X (2019). The deficiency of miR-214-3p exacerbates cardiac fibrosis via miR-214-3p/NLRC5 axis. Clin Sci (Lond).

[8] Wan H, Tian Y, Zhao J, Su X (2021). LINC00665 targets miR-214-3p/MAPK1 axis to accelerate hepatocellular carcinoma growth and Warburg effect. J Oncol.

[9] Wang J, Wang WN, Xu SB, Wu H, Dai B, Jian DD, Yang M, Wu YT, Feng Q, Zhu JH (2018). MicroRNA-214-3p: a link between autophagy and endothelial cell dysfunction in atherosclerosis. Acta Physiol (Oxf).

[10] Sang Z, Zhang P, Wei Y, Dong S (2020). miR-214-3p attenuates sepsis-induced myocardial dysfunction in mice by inhibiting autophagy through PTEN/AKT/mTOR pathway. Biomed Res Int.

[11] Yan Z, Zang B, Gong X, Ren J, Wang R (2020). MiR-214-3p exacerbates kidney damages and inflammation induced by hyperlipidemic pancreatitis complicated with acute renal injury. Life Sci.

[12] Li D, Liu J, Guo B, Liang C, Dang L, Lu C, He X, Cheung HY, Xu L, Lu C (2016). Osteoclast-derived exosomal miR-214-3p inhibits osteoblastic bone formation. Nat Commun.

[13] Zhang J, Li S, Li L, Li M, Guo C, Yao J, Mi S (2015). Exosome and exosomal microRNA: trafficking, sorting, and function. Genom Proteom Bioinform.

[14] Xie X, Xiong Y, Panayi AC, Hu L, Zhou W, Xue H, Lin Z, Chen L, Yan C, Mi B (2020). Exosomes as a novel approach to reverse osteoporosis: a review of the literature. Front Bioeng Biotechnol.

[15] Xiao P, Zhu X, Sun J, Zhang Y, Qiu W, Li J, Wu X (2021). Cartilage tissue miR-214-3p regulates the TrkB/ShcB pathway paracrine VEGF to promote endothelial cell migration and angiogenesis. Bone.

[16] Chen J, Yang Y (2021). LncRNA HAGLR absorbing miR-214-3p promotes BMP2 expression and improves tibial fractures. Am J Transl Res.

[17] Xin Z, Cai D, Wang J, Ma L, Shen F, Tang C, Hu L, Sun W (2020). MiR-214 regulates fracture healing through inhibiting Sox4 and its mechanism. J Musculoskelet Neuronal Interact.

[18] Sun W, Zhao C, Li Y, Wang L, Nie G, Peng J, Wang A, Zhang P, Tian W, Li Q (2016). Osteoclast-derived microRNA-containing exosomes selectively inhibit osteoblast activity. Cell Discov.

[19] Watson EC, Adams RH (2018). Biology of bone: the vasculature of the skeletal system. Cold Spring Harb Perspect Med.

[20] Fu R, Lv WC, Xu Y, Gong MY, Chen XJ, Jiang N, Xu Y, Yao QQ, Di L, Lu T (2020). Endothelial ZEB1 promotes angiogenesis-dependent bone formation and reverses osteoporosis. Nat Commun.

[21] Jiang X, Xu C, Shi H, Cheng Q (2019). PTH1-34 improves bone healing by promoting angiogenesis and facilitating MSCs migration and differentiation in a stabilized fracture mouse model. PLoS ONE.

[22] Oliviero A, Della Porta G, Peretti GM, Maffulli N (2019). MicroRNA in osteoarthritis: physiopathology, diagnosis and therapeutic challenge. Br Med Bull.

[23] Gargano G, Oliva F, Oliviero A, Maffulli N (2022). Small interfering RNAs in the management of human rheumatoid arthritis. Br Med Bull.

[24] Gargano G, Oliviero A, Oliva F, Maffulli N (2021). Small interfering RNAs in tendon homeostasis. Br Med Bull.

[25] Giordano L, Porta GD, Peretti GM, Maffulli N (2020). Therapeutic potential of microRNA in tendon injuries. Br Med Bull.

[26] Zhou LG, Shi P, Sun YJ, Liu HZ, Ni JQ, Wang X (2019). MiR-214-3p delays fracture healing in rats with osteoporotic fracture through inhibiting BMP/Smad signaling pathway. Eur Rev Med Pharmacol Sci.

[27] Altevogt P, Doberstein K, Fogel M (2016). L1CAM in human cancer. Int J Cancer.

[28] Angiolini F, Cavallaro U (2017). The pleiotropic role of L1CAM in tumor vasculature. Int J Mol Sci.

[29] Mrazkova B, Dzijak R, Imrichova T, Kyjacova L, Barath P, Dzubak P, Holub D, Hajduch M, Nahacka Z, Andera L (2018). Induction, regulation and roles of neural adhesion molecule L1CAM in cellular senescence. Aging (Albany NY).

[30] Drager O, Metz K, Busch M, Dunker N (2022). Role of L1CAM in retinoblastoma tumorigenesis: identification of novel therapeutic targets. Mol Oncol.

[31] Xie M, Huang P, Wu T, Chen L, Guo R (2021). Inhibition of miR-214-3p protects endothelial cells from ox-LDL-induced damage by targeting GPX4. Biomed Res Int.

